# Chromosome-Centric View of Genome Organization and Evolution

**DOI:** 10.3390/genes12081237

**Published:** 2021-08-12

**Authors:** Maria Sharakhova, Vladimir Trifonov

**Affiliations:** 1 Department of Entomology and Fralin Life Sciences Institute, Virginia Tech, Blacksburg, VA 24061, USA; 2 Department of Genome Diversity and Evolution, Institute of Molecular and Cellular Biology, SB RAS, 630090 Novosibirsk, Russia

Genetic material in all cellular organisms is packed into chromosomes, which represent essential units of inheritance, recombination, and evolution. Thus, our understanding of genome function is incomplete without knowledge of genome organization at the chromosome level. Recent advances in genome technologies, including long-read sequencing, chromosome flow sorting, Hi-C scaffolding, and optical mapping, allow the procurement of genome assemblies at the level of complete chromosomes. Physical genome mapping based on fluorescence in situ hybridization (FISH) enables direct correspondence of genomic sequences with particular chromosome bands or specific chromosome structures. Such chromosome-scale genome assemblies provide new opportunities for both cytogenetic and genome research. This Special Issue, entitled “Chromosome-Centric View of Genome Organization and Evolution” focuses on modern cytogenetic studies based on utilization of genome assemblies that are currently available for multiple organisms, including a key model organism, *Drosophila melanogaster*, and non-model organisms of vertebrates, insects, plants, and finally, humans. The issue contains twelve original research articles and 3 review papers devoted to recent achievements in the field of genomic studies of eukaryotic chromosomes.

The classical model organism *D. melanogaster* is represented in this Special Issue by two research articles and one review paper. The research article by Khoroshko et al. [1] describes the packaging of 15 genes with long introns in polytene chromosomes. The authors determined that such genes could occupy extended sections in polytene chromosomes containing band and interband series. The promoters of such genes localized in the interband fragments of the chromosomes whereas introns and exons formed additional independent bands. Some bands comprised both introns and exons while other bands contained intron material only. The research article by Volkova et al. [2] employs a combination of modern directed genome editing techniques, including the CRISPR/Cas9 system, to analyze activity regulation of the *Notch* gene, which is involved in cell differentiation in all multicellular organisms. The strongest phenotypic effects causing changes in *Drosophila* eye morphology were observed when combining the insertion with deletions that removed the sequence of the putative insulator of the gene. The review by Peterson et al. [3] discusses three-dimensional organization of the nucleus in *Drosophila* including chromosome folding into topologically-associating domains and chromosome territories within the nuclear space. This article provides a comprehensive picture of the multi-scale organization of the *Drosophila* genome.

Studies performed on a variety of vertebrates, including lizards, snakes, birds, and fish, investigated chromosome evolution, differences in recombination between sexes, and evolution of repetitive DNA. An article by Sassi et al. [4] describes chromosome and repetitive DNA evolution in eleven species from 4 genera of *Lebiasinidae* fishes. The study highlights different pathways of genome evolution between the genera. Three articles describe evolution of the sex chromosomes as an important part of the eukaryotic genome and whose evolution is often different from that of autosomes and varies across the animal taxa. These studies focus on a taxon squamata, in which chromosome evolution differs between families. Lisachev et al. [5] studied the content of the heterochromatic W chromosome of a lizard, *Eremias velox* (Lacertidae), and found a new repetitive sequence in this chromosome, which is not a microsatellite, as it was demonstrated for a number of non-recombining sex specific elements, but instead was part of an autosomal protein coding gene. Rovatsos et al. [6] describes a highly unusual system of multiple sex chromosomes in Madagascar chameleons of the genus *Furcifer*. Although, multiple sex chromosomes are very rare in species with female heterogamety, in the studied genus, such a sex-determination system looks to have been stable for at least 30 million years. In contrast to these studies, an investigation of ten species of pythons and boas conducted by Augustenova et al. [7] using conventional and molecular cytogenetic methods demonstrated that all examined species do not possess detectable sex-specific differences in their genomes indicating the presence of poorly differentiated sex chromosomes or the absence of sex chromosomes. 

Chromosome recombination during meiosis is an important component of sexual reproduction as it provides new recombinant sets of genetic information from parent to offspring. Malinovskaya et al. [8] analyzed differences in recombination rates between male and females of two swallow species that are distinguished by the presence or absence of sexual dimorphism. The study demonstrated elevated recombination rates in females with stronger sexual selection suggesting that such a mechanism could evolve as a compensatory reaction to runaway sexual selection in males.

Eukaryotic organisms developed different mechanisms to control the expansion of repetitive DNA in the genomes. Two articles in this issue are specifically devoted to the evolution of repetitive DNA as an important and understudied component of the eukaryotic genome. One of the most direct, albeit complicated, mechanisms programmed genome elimination or, so-called, chromatin diminution, has been described in some eukaryotic species. Timoshevskiy et al. [9] investigated genome elimination in the sea lamprey *Petromyzon marinus* and identified specific repetitive elements that mark 12 eliminated chromosomes in this species. The authors hypothesized that these sequences might target chromosomes for elimination and mediate the direction of their movement in chromosome elimination during anaphase. In another article by Biltueva et al. [10], the distribution of tandem repetitive elements in the polyploid sturgeon *Acipenser baerii* indicated that the polyploidization event was accompanied by repeat expansions. The authors employed repeated element-based specific probes to differentiate nascent paralogs resulting from recent whole genome duplication.

An increasing number of nearly complete and error-free genome assemblies in vertebrates generated new methodological challenges in understanding their genome evolution. The review by Iannucci et al. [11] describes novel technologies developed to bridge the gap between cytogenetics and genomics. One such approach relies on a single chromosome isolation and sequencing. The authors demonstrated key examples of applications of this approach for improving vertebrate genome assemblies and for better understanding the evolution of their chromosomes and karyotypes.

Chromosome studies based on chromosome-scale genomes may help to resolve puzzling questions in insect taxonomy and systematics and better understand the mechanisms of taxon diversification and speciation. The study by Naumenko et al. [12] determined genome-wide divergence in two cryptic malaria mosquito species, *Anopheles messeae* and *A. daciae*, whose taxonomic status is currently under debate. Genomic differentiation between the two taxa was especially pronounced on the inversion-rich X chromosome as well as by the frequencies of autosomal polymorphic inversions, suggesting a role for chromosomal rearrangements in species divergence. 

Studies in plants are represented by an article by Bielski et al. [13], which describes chromosome evolution in Old World lupins using comparative cytogenetic mapping based on FISH with oligonucleotide probes and chromosome-specific bacterial artificial chromosome clones. The results indicated that multiple changes in synteny between the lupin species, including putative translocations, inversions, and/or non-allelic homologous recombination, occurred during evolution and speciation events in this group of plants.

Finally, studies of human chromosomes in this Special Issue include a research paper and a review focusing on chromosome pathology. The research article, by Karamysheva et al. [14], describes a diagnostic of specific cases of chromosome abnormalities using in situ hybridization, microdissection, and multicolor banding in two cases of chromosome rearrangements in patients with balanced karyotype. This study provides recommendations for families who should be considered for genetic counseling. The review, by Tolmacheva et al. [15], analyzed the relationships between aneuploidy and methylation reprogramming in humans. The associations of these two phenomena highlight critical periods in ontogenesis and specific regions in the genome that play important roles in human reproduction and the formation of pathological phenotypes in newborns with chromosomal aneuploidies.

Overall, the papers in this Special Issue cover various aspects of genome organization and evolution at the chromosome level and demonstrate the necessity of applying different methods and developing new technologies to provide comprehensive insights into the dynamics and plasticity of the eukaryotic genome.
